# Inspection of Aircraft Wing Panels Using Unmanned Aerial Vehicles †

**DOI:** 10.3390/s19081824

**Published:** 2019-04-17

**Authors:** Vasileios Tzitzilonis, Konstantinos Malandrakis, Luca Zanotti Fragonara, Jose Angel Gonzalez Domingo, Nicolas P. Avdelidis, Antonios Tsourdos, Kevin Forster

**Affiliations:** 1 Centre for Autonomous and Cyber-Physical Systems, Cranfield University, Cranfield MK43 0AL, UK; Vasileios.Tzitzilonis@cranfield.ac.uk (V.T.); k.malandrakis@cranfield.ac.uk (K.M.); np.avdel@cranfield.ac.uk (N.P.A.); 2 Centre for Structures, Assembly and Intelligent Automation, Cranfield MK43 0AL, UK; j.a.gonzalezdomingo@cranfield.ac.uk; 3 Airbus UK, Broughton CH4 0DR, UK; Kevin.forster@airbus.com

**Keywords:** Non-Destructive Testing, ultraviolet light, automated inspection, defects detection, UAV, image processing

## Abstract

In large civil aircraft manufacturing, a time-consuming post-production process is the non-destructive inspection of wing panels. This work aims to address this challenge and improve the defects’ detection by performing automated aerial inspection using a small off-the-shelf multirotor. The UAV is equipped with a wide field-of-view camera and an ultraviolet torch for implementing non-invasive imaging inspection. In particular, the UAV is programmed to perform the complete mission and stream video, in real-time, to the ground control station where the defects’ detection algorithm is executed. The proposed platform was mathematically modelled in MATLAB/SIMULINK in order to assess the behaviour of the system using a path following method during the aircraft wing inspection. In addition, two defect detection algorithms were implemented and tested on a dataset containing images obtained during inspection at Airbus facilities. The results show that for the current dataset the proposed methods can identify all the images containing defects.

## 1. Introduction

In recent decades, the number of air passengers carried worldwide has increased by 78% [1]. Various sources [2,3] forecasts that in the next twenty years the world annual passenger and cargo air traffic to have an upward trend, with an annual growth rate at 4.5%. At the same time, the amount of post-production and maintenance Non-Destructive Inspection (NDI) or Non-Destructive Testing (NDT) is anticipated to increase in order to cope with the production levels. Motivated by this, global players from the commercial aircraft industry are designing new production lines or update existing ones, trying to accelerate the inspection process by utilising automated systems for heating, cleaning, chemical applications, etc. Currently, most of the inspection tasks are still carried out by a human operator. As a result, this might slow down the inspection chain. A promising solution for this challenge is the utilisation of automated systems, as proposed in [4], where an automated non-contact laser ultrasonic technology has the potential to increase both the annual revenue by up to 26.3% and the crack detection ratio from 44% to 95%. Recently, Unmanned Aerial Vehicles (UAVs) have attracted significant attention, for both military and civil applications, due to the advancements in processing power, miniaturisation of sensors and components that have led to an increase in the number of areas where they can be deployed. More precisely, a UAV could perform missions, such as search and rescue, disaster relief, surveillance, surveying, and so forth. Several researchers [5,6,7,8,9] have proposed the use of small UAVs for the inspection and monitoring of infrastructures such as buildings, wind turbines, photovoltaic systems, power transmission lines, and gas pipelines. In fact, the employment of UAVs for aerial inspection could minimise the risk of height hazard, inspection time and cost, since large areas under inspection are mainly assessed, in real-time, with minimal human interventions and without relying on any special infrastructure.

There is limited availability of research work in the aerospace sector for what concerns automated defect detection and more particularly in corrosion detection on metallic wing panels. Mumtaz et al. [10] proposed a method for crack detection in aircraft resorting to the use of a countourlet and discrete cosine transform. For what concerns the identification of corrosion, which is the main type of defect occurring in wing panels, Siegel et al. [11] propose a procedure relying on wavelets for characterizing and detecting the corrosion texture in aircrafts, while in [12] a watershed transform algorithm over the gradient of gray-level images is used. Ortiz et al. [13] propose a detector based on a three-layer feed-forward neural network to identify corrosion in large vessels. Another approach based on fractal properties is proposed by Xu and Weng in [14] for corroded surfaces. Finally, Zaidan et al. [15] focused their attention on the corrosion texture using the standard deviation and the entropy as discriminating features. All the above-mentioned methods are developed to identify defects under visual light and not using a UV light.

**Figure 1:**
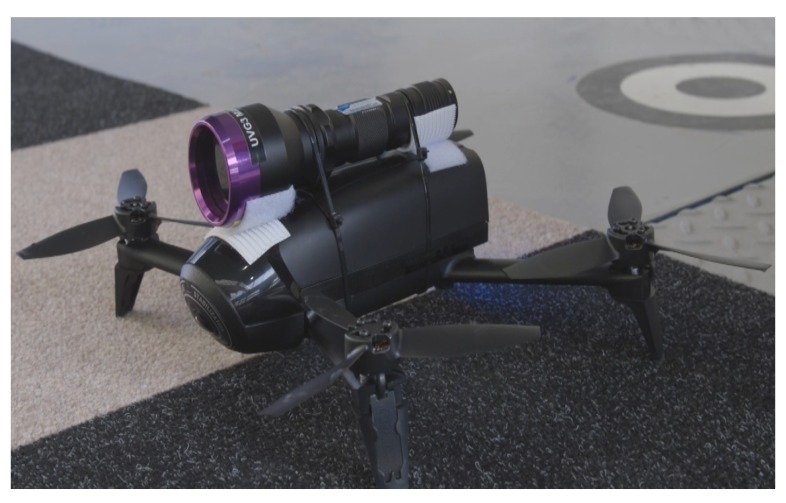
The aerial vehicle used in the proposed inspection methods.

Taking into consideration the aforementioned benefits of the aerial inspections, this work assesses the feasibility of using UAVs to automate the inspection of a metallic panel of an Airbus A320. Although research has been carried out on automated aerial inspection and maintenance on aircraft parts by using UAVs, no studies have been reported so far in the literature. In fact, the majority of the published papers, in this field, report the use of autonomous ground vehicles [16,17] or robotic arms for defects detection [18]. However, such autonomous systems might not be suitable for all the applications, because ground vehicles might not inspect high facilities, while robotic arms might require expensive equipment and appropriate infrastructure. In parallel, the main purpose of NDT is to check the airworthiness of a component without damaging it. In general, several NDT methods are available, including techniques such as visual inspections, borescope, liquid penetrant, eddy current, ultrasonic, acoustic emission, magnetic particle and radiography. For a comprehensive review, please refer to [19,20]. Among these NDT methods, the present study focuses on the visual and liquid penetrant inspection of metallic wing panels. In particular, is taking place prior to anodising, in order to check the wing panels for cracks and corrosion.First the penetrant is coated on to the part where capillary action pulls the penetrant in to any defect. Excess penetrant is washed off and the part is dried. Developer powder is the applied which absorbs an penetrant liquid left in the defect. Then under UV lighting the defect area is clearly visible. In this paper the automation of the final procedure of the defect recognition is proposed with the use of a small quadrotor (Figure 1), carrying a UV light, performing preprogrammed missions.

The obtained data are fed to the defect recognition algorithm for identifying possible defects. Results have shown that the propose system can identify defects even with the use of very limited data. The work presented in this paper covers the development of the complete system for aerial wing inspection, including the developed path following algorithm and the defect detection algorithm, and assesses their performance.

The paper is organised into five sections. In the following section, the selected aerial platform and the path following method for the aerial inspection are presented. The Section 2 describes the creation of the dataset. In the Section 3, the developed image processing method for the defects’ detection is analysed, while the scenario of the performed non-destructive testing is presented. Section 4 assesses the obtained results from the inspection and the fidelity of the system. In the last section, the significance of the findings is outlined, while future work on this study is suggested.

## 2. Hardware Selection and Path Following Method

**Figure 2:**
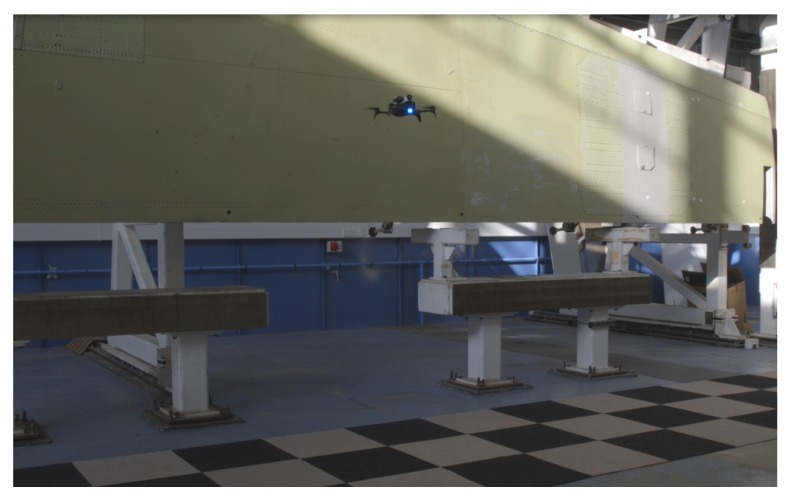
Aerial inspection.

In this section, an overview of the proposed UAV system is described. The setup mainly consists of the aerial vehicle, the Ground Control Station (GCS), and the wing panel for inspection. For the experiments a Bebop 2 was selected carrying a UV-light that fulfils the Airbus standard for the penetrant liquid inspection. The system can be seen in Figure 1. Detail information regarding the system can be found in [21]. For the testing a wing panel was utilised to examine the fidelity of the proposed aerial NDT methods. A 6 m long wing panel was vertically mounted to perform the visual and flow penetrant inspection methods, as shown in Figure 2.

**Figure 3:**
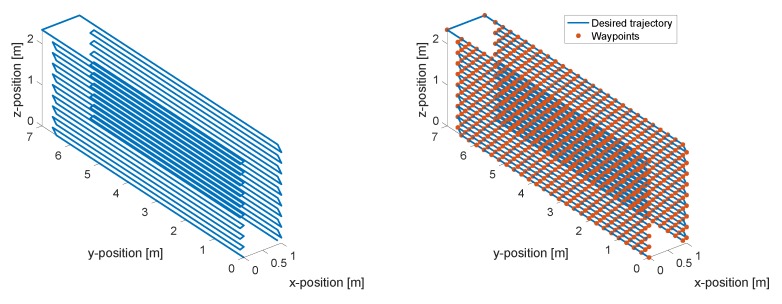
Desired waypoints and trajectory used for the simulations.

Figure 3 presents the waypoints and trajectory that need to be followed in the simulation of a two-sided inspection. The total inspection time for two-side wing panel was estimated at 1200 s, for a total approximately flight distance of 252 m. The velocity of the vehicle is considered constant. In the simulation model, a wing panel with 6.5 m span, 2.33 m chord, and 0.3 m thickness, is considered. The wing panel is assumed as a rectangular surface for simplifying its design complexity.

**Figure 4:**
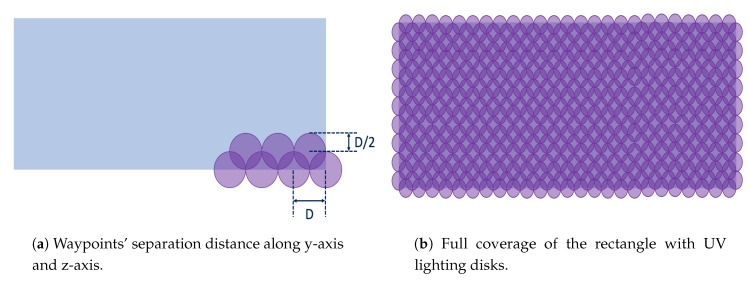
NDT simulated inspection.

The proposed inspection method is based on the full coverage of the rectangle with UV lighting disks, as is shown in Figure 4. The UAV keeps 1 m separation distance from the wing panel (x-axis) and hence the UV lighting diameter, which expresses the disks, is about 0.26 m. In the simulated mission, the UAV follows waypoints that have separation distance (D) 0.26 m along y-axis, 0.13 m in z-axis, as is shown in Figure 4a. An example of a wing panel that is completely covered by UV lighting disks is illustrated in Figure 4b. In this example, the number of waypoints for one-side inspection is set at 494 waypoints.

## 3. Dataset

**Figure 5:**
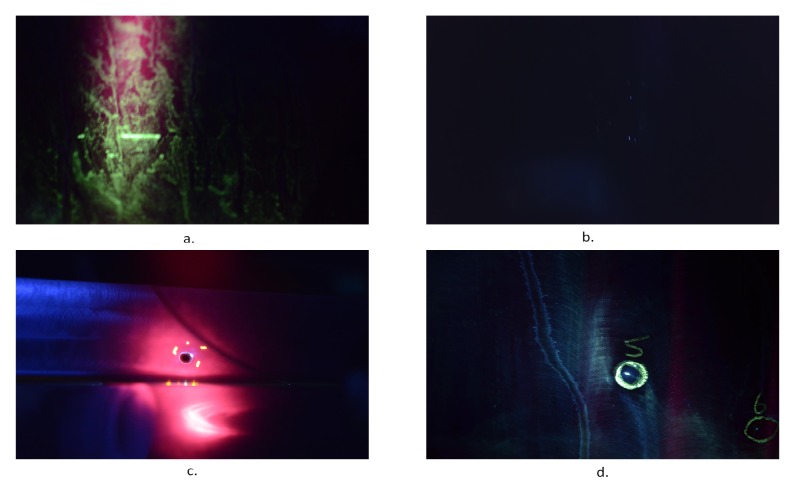
Images from the dataset (**a**) Image with green areas, (**b**) Image without green areas, (**c**,**d**) Images with possible defects.

The dataset which is used for validating the defect detection algorithms was created from images taken during inspection of wing panels in Airbus facilities. The photos were taken under UV light inside the black booth during an actual inspection. The equipment used was a DSLR camera of 24-megapixel and a mobile phone camera with 16-megapixel resolution. The dataset that was built has 119 images containing 25 images from 4 possible defects, images with green areas with no defects and images without any green. A representative sample can be seen in Figure 5.

**Figure 6:**
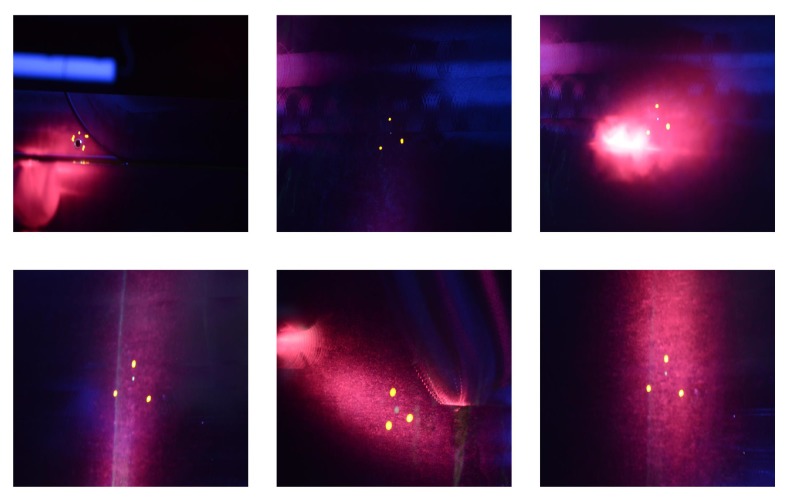
Baseline images with defects on wing panels.

Creating the dataset was a challenging task as it has to be done in parallel with the inspectors of Airbus in order to minimize the any possible disturbances in the production line. In addition, finding panels with defects was rare so the creation of the dataset took more than two months. However, the dataset is representative of the categories of findings during inspection. So it contains images with different lighting conditions under the UV light, images with glare like in Figure 5c or out of focus Figure 5a. In addition it contains images with green and non-green areas. This gives a more realistic representation of the images that will be obtained during drone inspection and also to tune the algorithms to work under different image quality data. From the 25 images containing defects, 11 were used as a reference images. All the images of the dataset were compared with those to see if they contain any defect. These reference images were referred as the baseline images and a sample of them can be seen in Figure 6. These 11 images visualize the defects from different angles, with glare or are out of focus, with limited light etc. The selection was made after examining the dataset and deciding to have the most representatives images regarding the different conditions mentioned above.

## 4. Defect Recognition Algorithms

**Figure 7:**
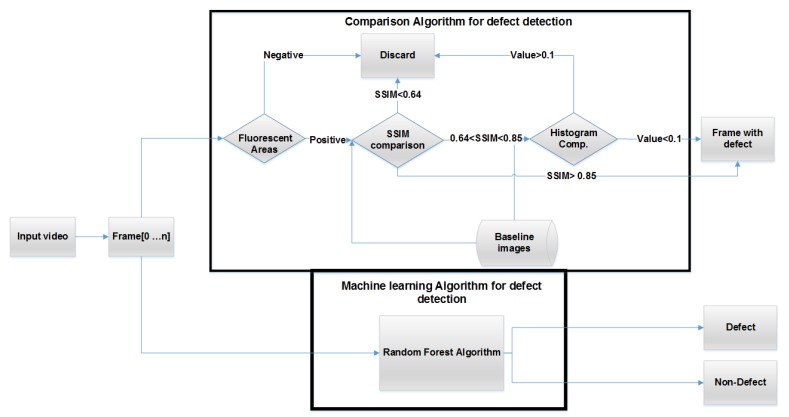
Flowchart of both the proposed defect recognition methods, the one with the combine use of SSIM and Histogram comparison and the Random Forest.

Some of the challenges that the algorithms had to address was the conditions of lighting and how to distinguish the fluoresce areas with defects with the ones without defect. During the inspection most of the areas that luminance under UV light with green do not contain any defect. To address the problem, two different approaches were implemented and tested. In the first one, images with defects were compared with image data from inspection and their similarity was calculated by combining two different image processing algorithms. In the second, a Random-Forest algorithm was implemented for classifying the images in defect or not categories. The results of both methods were compared. In Figure 7 the flowchart of the proposed methods can be seen.

In the first method, the software initially filters the images keeping only the ones that contain green areas. Then it uses two different algorithms, Structural Similarity Index Measure [22] and Histogram Comparison, to classify if these images contain any possible defects. In a step by step description of Figure 7 the algorithm first discards any images that do not contain any green area. Then the remaining images are compared with the baseline images using the Structural Similarity index Measure (SSIM) [22]. The outcome of this comparison can be either positive (defect was found), negative (no defect) or further investigation is needed. The outcome depends on the different values of the SSIM. If further investigation is needed a histogram comparison with the baseline images is taking place. In the second method the images are fed to a Random Forest Classifier. Detail description of both methods is given in the rest of the section.

**Figure 8:**
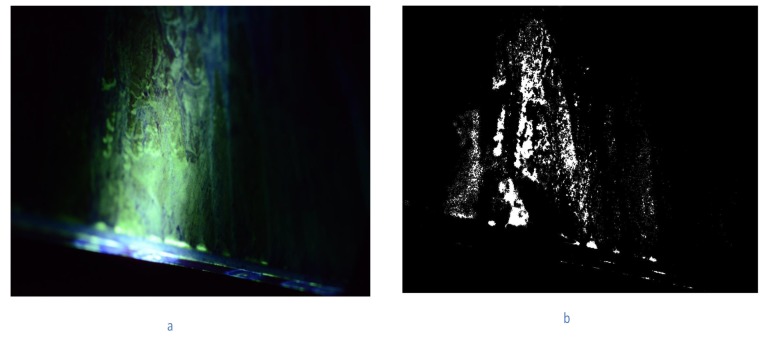
(**a**) Initial Image containing green areas, (**b**) Image after masking.

For the first method, the initial step is to clean the data, by excluding all the images that do not contain any green areas (fluoresce liquid). This is necessary because the biggest area of the wing panel will not have any fluoresce liquid during inspection so most of the data obtained during automated imaging inspection do not have any green in it. If these data were processed in the next steps of the software a lot more of computational time will be needed. So these data are necessary to be discarded. For this reason, a mask was implemented. The mask covers all the pixels that do not have value in the range of green in the RGB and colours with white the green ones (Figure 8).

**Figure 9:**
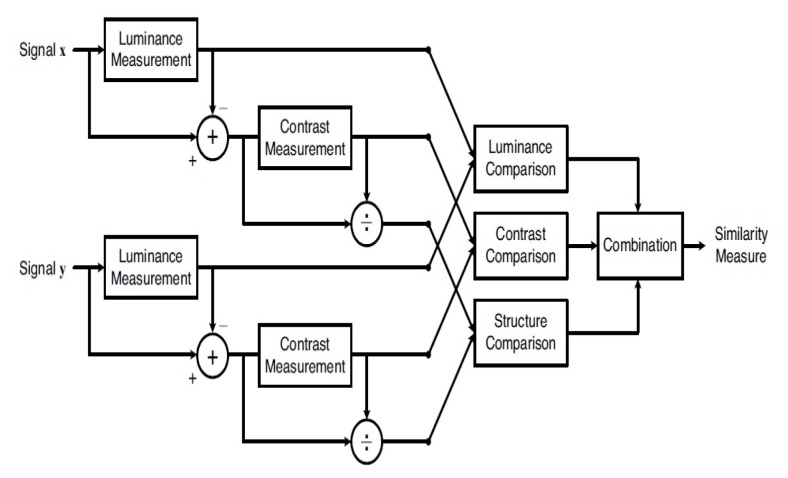
Diagram of the structural similarity (SSIM) measurement system.

Then the algorithm finds the contours in the images. Contours can be explained as the closed curve joining all the points delimiting an area with the same colour or intensity. If the image contains at least one contour meaning that then definitely has some green colour. After filtering, the remaining images containing green areas are compared with the baseline images that were taken at Airbus facilities. For the comparison, the algorithm of SSIM was used. SSIM is an index used for measuring the similarity of two images. It can be seen as a quality measure of the image that is being compared and it takes values between −1 and 1. If the compared image is completely different from the initial the value will be −1 while if they are identical/very similar it will be 1. The comparison is made by measuring luminance, contrast and structure. Image Structure is an arrangement and organization of interrelated elements in an image. The structural diagram of the algorithm is presented in Figure 9.

If the SSIM value of the compared images is between 0.64 and 0.85 then the images are further compared using a histogram comparison algorithm. A histogram represents the distribution of pixel intensities (whether colour or greyscale) in an image. It can be visualised as a graph (or plot) that gives a high-level intuition of the intensity (pixel value) distribution. As the colour identification is very important in this task, image descriptors that characterise the colour of an image are needed. Histograms can model the distribution of the pixel intensities in each colour channel of the image and are thought to be very powerful images descriptors used in image search engines and as feature extractors in different machine learning algorithms. Each baseline image’s histogram is calculated for only the green channel and then compared with the histogram obtained from the image from the inspection. Then the histograms of the images were compared with the histograms of the baseline. Four different algorithms of histogram comparison were tested, Correlation, Chi-Squared, Intersection and Bhattacharyya distance for finding the one best fitter in our dataset. The algorithms were implemented with the use of OpenCV [23] and the algorithm with the best result for the dataset was Bhattacharyya distance. If the outcome value of the comparison is below 0.1 then the image can be classified as an image with a defect. The thresholds of both the algorithms were set after experiments on the data. In the second method a Random Forest algorithm was implemented, trained and tested on the existing data. Random Forest’s need relatively little data compared with Deep Learning algorithms (CNN, etc.). In addition, usually, the outcome is a robust model. The features that the algorithm was trained with were colour and texture.

## 5. Results and Discussion

The flight simulation results indicate that the non-linear guidance law (NLGL) provides satisfactory performance. More precisely, the complete inspection of a whole panel, using NLGL, lasted 1260 s that corresponds to less than 5% discrepancy from the estimated.

The results of the actual aerial inspections indicated that the UAV can carry out successfully both visual and flow penetrant inspections. During the visual inspection, the aerial vehicle has shown a robust and stable behaviour using the optical flow algorithm. The UAV could land within ±0.15 m (for both x and y-axis) from the defined landing point. It was observed that the ambient light of the inspection room significantly affected the performance of the optic flow algorithm and hence the vehicle’s behaviour and performance, giving room for further studies about localization using another set of sensors (e.g., laser, ultrasonic or infrared).

**Figure 10:**
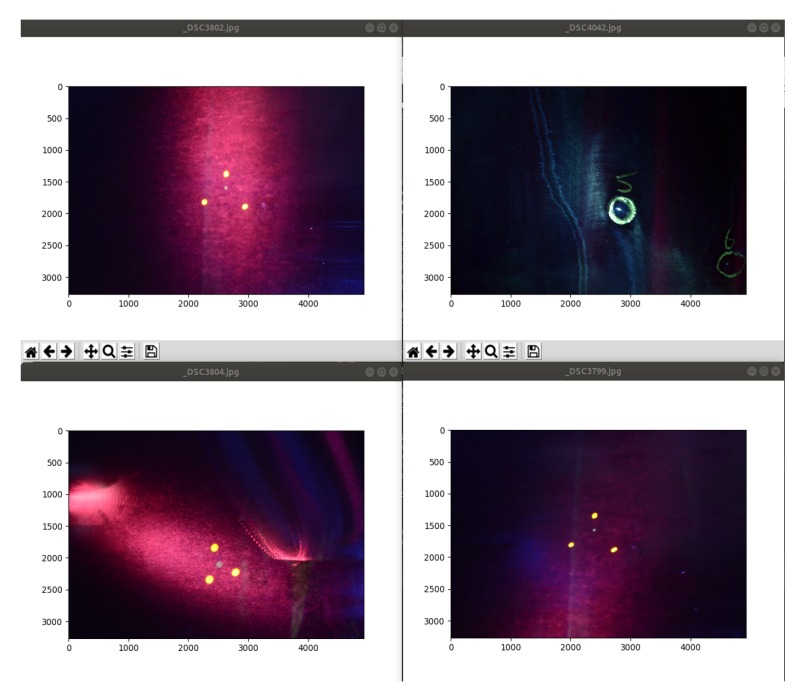
Images identified with defect using the combined SSIM and Histogram comparison method.

Regarding the defect detection methods, both were tested on the dataset created during inspection and described above. The results for the first method are very convincing as the software manages to identify all the 25 images containing defects on the specific dataset plus one image with a possible defect. Considering the limited number of images with defects the performance is very good. Images with defects displayed by the software output can be seen in Figure 10.

**Figure 11:**
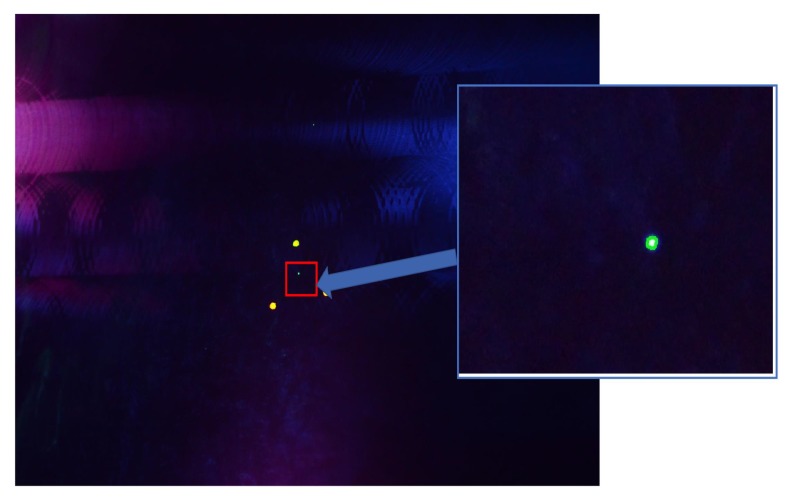
Image with possible defect.

**Figure 12:**
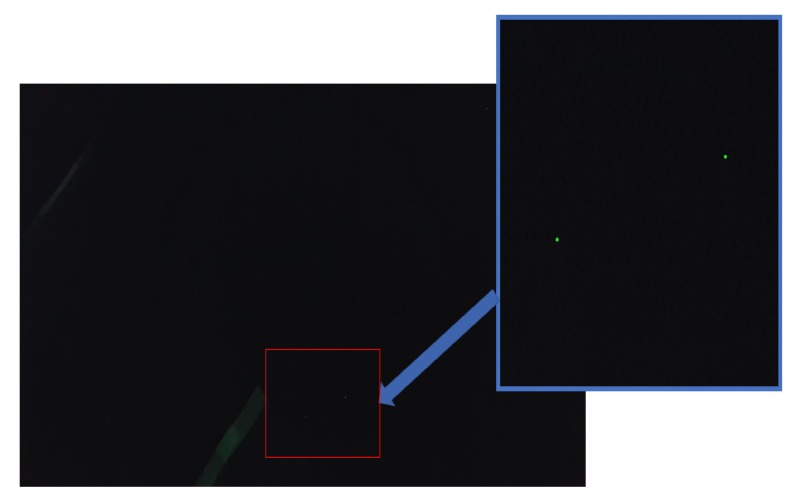
Image with false positive defect.

In Figure 11 and Figure 12 two images with possible defects can be seen. Image in Figure 11 is one of the 25 images containing possible defect and in Figure 10 is an image without any defect but contains green spots. The images are very similar so the software has classified it as a possible defect. This is due to the limited amount of images containing defects but as more images will be added to the dataset the performance of system will be improved.

For training and testing the Random Forest algorithm the whole dataset was used plus some pictures collected inside the inspection room showing images of the environment (i.e., exit room, floor, walls, etc.). The total number of representative images used to train and test the Random Forest algorithm was 134. The dataset was labelled using two classes: defect and non-defect. After the labelling, 10 images were randomly selected from the dataset prior to training be used as a test set. Thus, the test set comprises totally 10 images of which 4 belonged to the defect class and 6 to the non-defect class. Finally, the remaining dataset was split into a training and validation set. The ratio used was 80% for the training set and 20% for the validation set, as commonly used for small data set [24]. The model even with this amount of data had promising results with overall accuracy on the validation data of 97%. The result of the validation can be seen in Table 1.

The metrics were produced with the use of sklearn.metrics [25]. The precision is defined by Equation (1) where tp is the number of true positives and fp the number of false positives. The precision is intuitively the ability of the classifier not to label as positive a sample that is negative. Recall is defined by Equation (2) where tp is the number of true positives and fn the number of false negatives. The recall can be said that is the ability of the classifier to find all the positive samples.

(1) tp÷(tp+fp)

(2) tp÷(tp+fn)

(3) F1=2×(precision×recall)/div(precision+recall)

*F*1 can be described as a weight average of the precision and recall and it is given by Equation (3). *F*1 best and worst values are 1 and 0, respectively. The relative contribution of and precision and recall to *F*1 are equal. In a multi-class case *F*1 is the weighted average of each class. Support is the number of each class in the validation data. From Table 1 it can be said that the model has the ability to find all the positive samples in the defect category while the precision in the same category is relative low 0.86 meaning that the model recognises some false positives. This can be explained due to the limited number of images with defects. For the non-defect category where the number of images was much larger than the ones with defects, 109 non-defect vs. 25 with defect, the results are better with precision of 1 in the validation data.

**Figure 13:**
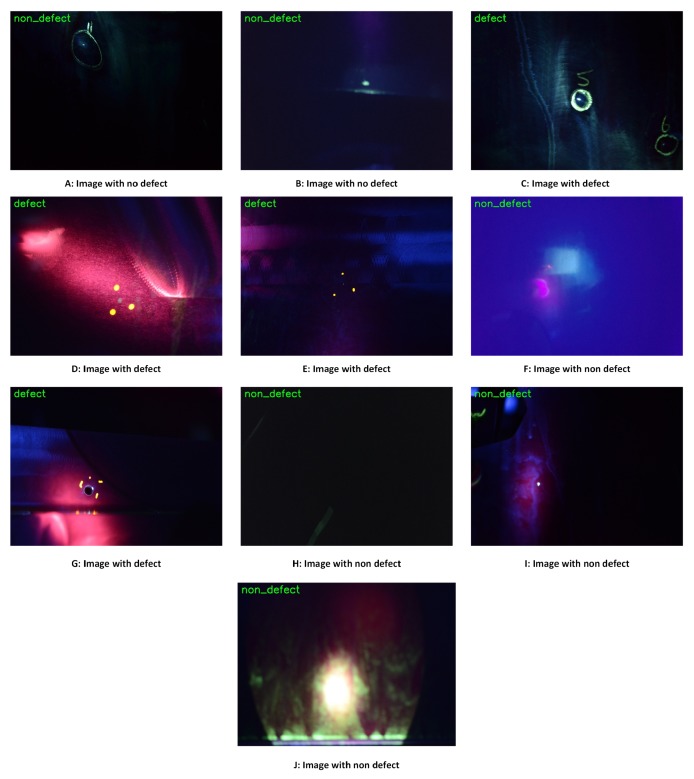
Results of the Random Forest algorithm. (**A**–**J**) titles are the class were the image actually belongs and the green title on the image is the outcome of the Random Forest algorithm.

The model was tested also in 10 randomly selected images from the dataset. The accuracy of prediction was 100% and the results can be seen in Figure 13.

Results show that both defect methods are performing very well in identifying defects. It is worth noticing that the image in Figure 12 that was classified as false positive in the combined SSIM and Histogram method, with Random Forest was classified correct, labelled image H in Figure 13. The comparison method is performs well but the computational time needed for running the whole dataset was 3 min in Intel Xeon 2.66 GHz processor, so as the dataset will get larger it will be even slower as more comparisons will be needed to be performed. On the other hand, testing and validation results of Random Forests are promising and indicate that this approach is able to detect defects even with this small dataset. In addition, the Random Forest algorithm performs faster than the comparison method. The random forest algorithm classified the 10 random images in 5 s which is significantly less than the comparison method which needs 15 s for the same number of images. Though it is not possible to extract solid conclusions in such a small dataset, the machine learning method has an advantage, as once properly trained will be able to generalize better on the data rather and will also will be faster than the comparison method.

## 6. Conclusions

This study objectives were to study the feasibility of replacing a standard NDT procedure, in a real operational environment, by performing the task using an automated aerial inspection thus reducing the overall risk, time and economical costs. This research shows how a commercially available UAV carrying a UV light integrated with an image processing algorithm can provide an easy-to-implement and robust method for visual and flow penetrant inspection. Simulation tests, in which a NLGL is modelled on the UAV, confirm reasonable tracking performance for the aerial wing inspection. Furthermore, experimental results show that the proposed components and architecture can offer additional capabilities to the operator such as automated record-keeping. For what concerns the defect recognition, two different image processing algorithms were implemented and tested using precious real inspection data. The first algorithm combined a two-step image processing procedure: a threshold-based classification using the SSIM and a histogram comparison. This two-step image processing was not tested before, to the best of the authors knowledge. The second algorithm implements a machine learning approach using a Random Forest classifier. Both algorithms performed very well on the available dataset, with accuracy reaching up to 97%. SSIM and Histogram approach is able to perform well for the specific dataset but once the dataset grows significant more computational will be need to perform the comparisons with the baseline. On the contrary, Random Forest is faster in predicting the outcome and less depending on the amount of data available, as less samples are required for training. Results from both methods were evaluated and validated by qualified NDT Airbus inspectors. Additional work could be performed on the creation of a more reliable dataset. This would require a considerable amount of time (in the order of months of inspections), as real defects are seldom found during inspections. Once a large dataset exists, different machine learning classifiers can be trained, validated and tested. Furthermore, different path following methods could be studied for determining the most appropriate guidance law for aerial inspection. Further experimental aerial inspections could be carried out including the studied path following method to validate the behaviour and performance of the simulation model.

## Figures and Tables

**Table 1:** Random Forest Validation Results.

Category	Precision	Recall	*F*1-Score	Support
Defect	0.86	1.00	0.92	6
Non-defect	1.00	0.95	0.97	16
Avg/Total	0.97	0.96	0.96	25

## Abbreviations

The following abbreviations are used in this manuscript:

NDI	Non-Destructive Inspection
NDT	Non-Destructive Testing
UAV	Unmanned Aerial Vehicle
UV	Ultraviolet
GCS	Ground Control Station
NLGL	Non-linear Guidance Law
LOS	Line-of-sight
LQR	Linear Quadratic Regulator
DSLR	Digital Single Lens Reflex
SSIM	Structural Similarity Index Measure
